# The predictive power of teaching self-efficacy and emotion regulation on work engagement for EFL teachers in higher education

**DOI:** 10.3389/fpsyg.2025.1446257

**Published:** 2025-06-17

**Authors:** Yan Wang, Lin He, Qing-Yi Wang

**Affiliations:** ^1^School of International Programs, Xi’an International Studies University, Xi’an, Shaanxi, China; ^2^School of English Studies, Xi’an International Studies University, Xi’an, Shaanxi, China; ^3^School of Basic Medicine, Fourth Military Medical University, Xi’an, Shaanxi, China

**Keywords:** EFL, teaching self-efficacy, emotion regulation, work engagement, higher education

## Abstract

**Introduction:**

The study aims to explore the role of emotion regulation and teaching self-efficacy in predicting work engagement among Chinese English-as-a-Foreign-Language (EFL) teachers in the higher education sector.

**Methods:**

A quantitative approach was employed to conduct this research. Three validated questionnaires were distributed to Chinese college EFL teachers with diverse experiences and academic backgrounds. A total of 495 teachers completed the questionnaires. The questionnaires assessed emotion regulation, teaching self-efficacy, and work engagement. Correlational analyses were initially performed to examine the relationships among these variables. Additionally, structural equation modeling (SEM) was applied to further investigate the predictive power of teaching self-efficacy and emotion regulation on work engagement.

**Results:**

The correlational analyses showed a strong association between emotion regulation, teaching self-efficacy, and work engagement. The results of the structural equation modeling (SEM) indicated that teaching self-efficacy significantly and favorably predicted work engagement among Chinese college EFL teachers. In contrast, emotion regulation did not show obvious contributing power towards work engagement in Chinese higher education EFL context.

**Discussion:**

The findings reflect unique features of Chinese college EFL teachers’ work engagement and provide useful implications for college EFL teachers and educational authorities in that it is suggested more time and energy should be invested in facilitating teachers with their psychological and emotional well-being along with pedagogical concerns.

## 1 Introduction

Teachers are constantly exposed to various challenges in the classroom teaching context and are believed to be the most critical part in the educational systems and the central pillars of society (Chu, 2021). A majority of teachers are extremely passionate about their career and engrossed in the education process whole-heartedly. Such enthusiasm and commitment are termed as work engagement, which is “a positive, fulfilling and work-related state of mind that is characterized by vigor, dedication and absorption dimensions” (Schaufeli et al., 2002, p.202). In most cases, high levels of teachers’ work engagement impose a favorable influence on teaching quality and enhance students’ performance. Teachers who are engaged in their jobs are more likely to take initiatives in the face of challenge and be more committed to and passionate about their work. It had been found out that engaged educators are not only more prone to add involvement in school life but also assume extra responsibilities outside the class (Chen, 2024). Since work engagement is such a pivotal determinant of learner performance and is closely related to educators’ productiveness, several scholars are beginning to focus on it and have testified various contributing factors to the EFL teachers’ work engagement (Greenier et al., 2021).

To unveil the mechanism of work engagement, emotion regulation and teaching self-efficacy play relevant roles. Emotion regulation is a crucial aspect of a teacher’s professional life and is regarded as a potential predictor of teacher work engagement (Gross and John, 2003). As Gross and John (2003) elaborated in their study, emotion regulation relates to “various cognitive, physiological, and behavioral processes that a person employs to regulate his or her emotional expressions and experiences.” According to a study by Li (2023), teachers who efficiently navigate their emotions are more successful in classroom management, discipline, and their relationships with students. Greenier et al. (2021) found that teachers who can down-regulate the negative feelings and up-regulate the positive ones are more possibly to succeed in their careers. The findings are supported by another study done by Ma and Lenz (2023), which asserted that language teachers who maintain healthy emotional control tend to be more engaged and determined in their work. The study also found that language teacher emotion regulation could positively and significantly predict teachers’ self-efficacy beliefs and engagement at work.

Along with emotion regulation, teaching self-efficacy is another frequently researched field in terms of factors influencing teachers work engagement. The concept of self-efficacy originates from the foundation of social cognition, emphasizing the idea that people can influence their own agency (Bandura, 2006). It addresses the fact that individual’s behavior can affect their intended performance and in turn influence final achievements. In the situation of teaching, the concept of self-efficacy relates to their perception of their own capability in terms of managing the classroom teaching, supervising students’ performance and manifesting targeted teaching tasks (Tschannen-Moran and Hoy, 2001). Teaching self-efficacy contributes to many aspects in the teaching process, including determining educators’ personal goals, influencing the degree of their perseverance in adversity and motivating their drive to carry out certain teaching behaviors such as utilizing digital resources. Realizing the significance of teaching self-efficacy, many research in EFL context have examined its impact on various psychological constructs such as job satisfaction, emotional intelligence, identity, burnout (Namaziandost et al., 2023; Shen, 2022).

Researchers have paid great attention to various factors contributing to teachers’ work engagement, among which emotion regulation and teaching self-efficacy were considered as two essential elements (Derakhshan et al., 2023; Ma, 2023). In a recent survey conducted by Chen and Tang (2024), 410 Chinese EFL teachers were chosen to participate in a quantitative study regarding the interconnection among emotion regulation, well-being and work engagement and the results specified that 65 percent of changes in the EFL educators’ engagement can be predicted by their well-being, and about 73 percent can be predicted by their emotional regulation. In another study done by Johnson (2022), teachers’ self-efficacy was evaluated to see if it predicted teacher work engagement for expatriate teachers in international schools in China. The findings indicated a statistically significant predictive relationship between teachers’ self-efficacy and teacher work engagement.

Though these studies have provided some understanding of how these psychological factors affect EFL teachers’ work engagement, the data reported so far are mostly obtained from elementary and secondary education context (Shu, 2022). More importantly, the lack of research on teachers’ emotion regulation and work engagement in higher educational contexts appears to be even more rampant in China. After perusing existing literature in China National Knowledge Infrastructure (CNKI), it was surprising to see that there were less than 30 papers focusing on this issue, within the last 5 years. This gap in research is notable given the unique cultural, societal, and educational dynamics that characterize China’s educational system. The scarcity of such research limits the development of targeted interventions and strategies that could enhance teacher engagement and in turn improve teaching effectiveness and student achievement. According to King and NG (2018), it is imperative that future research endeavors to address this void to foster a deeper understanding of the factors influencing teacher engagement in China and to inform policy and practice that can lead to more vibrant and effective educational environments.

**Figure 1:**
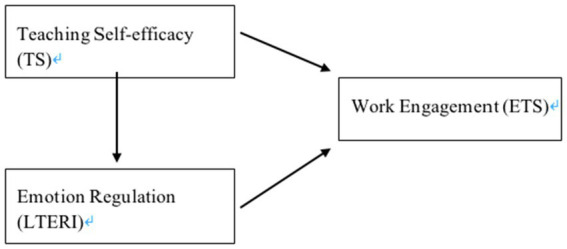
Conceptual framework.

Therefore, in line with and to supplement the previous research, this study focuses on educators teaching English as a Foreign Language (EFL) in contexts where English was neither widely used for communication nor used as the medium of instruction, and aims to examine the predictive role of teaching self-efficacy and emotion regulation on work engagement. A model was proposed in the current research (Figure 1).

Then, this proposed model was tested via Structural Equation Modeling (SEM) employing AMOS 21.0. The following research questions were put forward to meet these purposes:

1. Are there any significant correlations between Chinese college EFL teachers’ emotion regulation, teaching self-efficacy and work engagement?
2. To what extent can teachers’ work engagement be predicted by emotion regulation for Chinese college EFL teachers?
3. To what extent can teachers’ work engagement be predicted by teaching self-efficacy for Chinese college EFL teachers?

Consistent with the research questions, the following hypotheses were proposed:

*H1*: Teaching self-efficacy significantly contributes to emotion regulation for Chinese college EFL teachers.

*H2*: Emotion regulation significantly contributes to work engagement for Chinese college EFL teachers.

*H3*: Teaching self-efficacy significantly contributes to work engagement for Chinese college EFL teachers.

## 2 Literature review

### 2.1 Work engagement

Derived from positive psychology, work engagement is recognized as a motivational construct and is defined as “a positive, fulfilling, work-related state of mind that is characterized by vigor, dedication, and absorption” (Schaufeli et al., 2009). Vigor refers to one’s psychological willingness and physical vitality to invest efforts into work in adverse or challenging conditions. Dedication pertains to the enthusiasm and engagement one displays in the procedure of work for the purpose of achieving targeted outcomes. Absorption is regarded as a mindset to which an individual is so addicted and is completely immersed in the working activities. The Work Engagement Theory (WET) indicates an engaged person enjoys a positive attitude represented through endless vitality, energy and determination to attempt and invest time and effort in a target. As Klassen et al. (2012) discussed in their research, teachers’ work engagement is regarded as a multidimensional motivational construct and is characterized by the voluntary sharing of their physical, cognitive and emotional resources in classroom teaching.

Many academics began focusing on various positive aspects of work-related health outcomes, particularly work engagement, after the recent positive psychology movement (Dewaele et al., 2019; Wang et al., 2021). Xiu (2024) looked into what influences pre-service teachers’ involvement in their jobs. The necessary information was obtained from 2,247 Chinese teachers and principals through surveys. The findings showed a strong correlation between teachers’ work engagement and school cultural climate as well as job resources. Minghui et al. (2018) looked at the relationship between social support and teacher efficacy and work engagement in a different study. To achieve their goal, three trustworthy questionnaires containing the constructs were distributed to 1,027 Chinese teachers. The researchers discovered a relationship between teacher efficacy, social support, and work engagement based on the findings of correlation analysis. Parallel to this, Van Der Want et al. (2019) investigated the potential relationship between the identity, self-efficacy, and work engagement of teachers. Twenty-nine teachers were chosen from various Dutch schools to work toward this goal. To get participant opinions on the relationship between identity, self-efficacy, and work engagement, the researchers used questionnaires and semi-structured interviews. After examining the replies, the researchers concluded that teachers’ identities and levels of self-efficacy pose significantly positive effect on the work engagement of teachers. Greenier et al. (2021) gathered data utilizing a sequential explanatory mixed-method approach and compare the results among British and Iranian teachers. The findings concluded that emotion regulation and psychological well-being significantly predicted teacher work engagement in the context of EFL teaching, and the association between psychological well-being and work engagement was stronger for British teachers. Align with this study, Derakhshan et al. (2023) investigated the association among loving pedagogy, teaching for creativity, and work engagement for multinational EFL teachers and their findings shows that loving pedagogy and teaching for creativity significantly predicted work engagement for teachers.

Despite the above-mentioned literature focusing on antecedents of work engagement, a limited number of research probes this issue in the domain of higher education. In a recent study carried out by Wang and Pan (2023), using Structural Equation Modeling (SEM), teacher self-efficacy and resilience were examined to testify their predictive power for work engagement in Chinese higher education context. The researchers concluded that both teacher self-efficacy and resilience could significantly predict EFL teachers’ work engagement while teachers’ self-efficacy served as a stronger predictor than resilience. Regardless of these aforementioned studies, there is comparatively less literature focusing directly on the investigation of antecedents of teacher engagement, particularly in the field of higher education and EFL teaching. Hence, this study attempts to compensate for this scant attention by examining the possible predictive roles of teachers’ self-efficacy and emotion regulation in the work engagement of university EFL teachers in China.

### 2.2 Teaching self-efficacy

Derived from the social cognitive theory (Bandura, 1977) and Rotter’s social learning theory (Rotter, 1966), efficacy pertains to an individual’s expertise and competencies to attain certain goals or desired achievements (Bandura, 1977) and the concept is explicitly defined by Bandura (1986) as “people’s judgments of their capabilities to organize and execute courses of action required to attain designated types of performances” (p. 391). With respect to teaching context, self-efficacy refers to teachers’ judgment of their abilities in organizing the classroom, engaging students and performing expected teaching objectives (Tschannen-Moran and Hoy, 2001), and teachers’ efficacy is considered as the recognition of their capabilities to achieve desired targets even among unmotivated learners (Tschannen-Moran and Hoy, 2007).

It is worth noting that self-efficacy is a motivational construct depending on one’s own perception of capability rather than actual level of competence. Therefore, maximum performance would be achieved when teachers slightly overestimate their real professional skills because they are more motivated and willing to expend their efforts and take the full advantages of the capabilities they do possess in a challenging situation (Tschannen-Moran et al., 1998). To be more specific, greater self-efficacy in teachers leads to higher levels of engagement in demanding activities, increased effort and resilience in the face of difficulties, and enhanced commitment to the learning objectives (Fathi et al., 2020).

Bandura (1977) categorized self-efficacy into two dimensions: personal efficacy and outcome expectancy, respectively referring to the belief about one’s own capabilities and that one’s behavior will result in optimal outcomes. Put another way, teachers with both strong confidence in their competencies and affirmed belief in desirable teaching outcomes are more likely to be dedicated in the teaching process. Another three-dimensional classification of teachers’ self-efficacy was introduced by Tschannen-Moran and Hoy (2001), namely efficacy in classroom management, efficacy in student’s engagement, and efficacy in instructional strategies. Efficacy in classroom management relates to teachers’ confidence in building in-class discipline and monitoring the classroom activities. Efficacy in student’s engagement refers to teachers’ beliefs of their inspirational abilities in encouraging students’ active participation in classroom tasks. Efficacy in instructional strategies pertains to teachers’ perception of their competence to manoeuvre effective teaching strategies so as to attain desirable learning outcomes.

Acknowledging that teaching self-efficacy is a prominent construct affecting on educators’ choice of individual purpose as well as their intended performance, many researchers have conducted investigations on its diversified psychological associations and impacts such as job satisfaction, burnout, commitment, identity, work engagement to name a few (Wang et al., 2021; Han and Wang, 2021). Many researchers have pointed out the positive correlation between teaching self-efficacy and commitment (Fathi et al., 2020; Shu, 2022). Teachers with higher level of efficacy are inclined to adopt creative teaching strategies and invest more efforts to achieve the greatest work potentiality (Liu et al., 2021). Their findings also indicate that teaching self-efficacy had an essential function in determining key scholastic result in the career setting. On the opposite side, research has demonstrated that burnout is negatively associated with teaching self-efficacy, which means teachers with higher level of self-efficacy are relatively less prone to burnout (Chen et al., 2024).

Han and Wang (2021) gathered data from 614 Chinese EFL teachers with various experiences and academic degrees by distributing questionnaires and examined the correlation among self-efficacy, work engagement and reflection. The findings showed that the three constructs were positively correlated and teachers’ self-efficacy and work engagement significantly predicted their reflection. In another study in different social context, a multi-level analysis was conducted with 96 Swiss vocational teachers in orders to examine the interrelations between teachers’ self-efficacy, responsibility and students’ engagement. The results suggested that teachers’ self-efficacy predicts their autonomy-supportive teaching, which in turn is a strong propeller for student engagement (Lauermann and Berger, 2021).

### 2.3 Emotion regulation

Since its emergence in the late 1990s, emotion regulation had caught the attention of many education psychologists and the concept is defined in various terms. For instance, Cole et al. (1994) defined emotion regulation as “the ability to respond to the ongoing demands of experience with the range of emotions in a manner that is socially tolerable and sufficiently flexible to permit spontaneous reaction as well as the ability to delay spontaneous reaction as needed” (p.74). For Gross (1998), emotion regulation is a combination of a variety of process in with the aim of controlling when and how people experience and express their emotions.

According to Sutton and Harper (2009), emotion regulation is categorized from two perspectives: one is emotion up-regulation, which is unitized to strengthen one’s emotion and the other is emotion down-regulation focusing on weakening or controlling some emotional incidents. In the context of teaching, teachers are experiencing waves of diverse emotions that needs to be properly regulated as teacher emotions are considered as a critical factor of building classroom climate which affects students’ interest in and motivation for learning (Frenzel et al., 2021). These emotions include not only positive ones such as happiness, joy, pride and enthusiasm, but also negative ones such as anger, anxiety, frustration, shame and disappointment (Yang et al., 2021). To formulate a nurturing learning environment and enhance the teaching effectiveness, teachers are expected to employ emotion regulation strategies in the classroom environment, i.e., express their positive emotion such as joy and pride and hinder their negative emotions such as anger and frustration (Frenzel et al., 2024).

Another theory proposed by Gross (1998) was the process-oriented model of emotion regulation, which comprehensively characterized emotion regulation into five strategies, namely situation selection, situation modification, attention deployment, cognitive change and response modulation. In contrast to the last component, which is used to modulate the effects of fully developed emotional responses (response-focused strategies), the first four processes are antecedent-focused strategies because they are used before complete emotional response activation. Greenier et al. (2021) further defined antecedent-focused strategies as “commonly used by teachers before the initiation of the emotional arousal stages” while response-focused strategies are “typically employed by teachers after the initiation of the emotional arousal stages.” Existing literature on teacher emotion regulation reflected different strategies adopted by teachers, among which includes self-awareness and self-regulation as well as suppression (Heydarnejad et al., 2021).

In the field of language teaching, EFL teachers are more inclined to experience negative emotions such as anxiety and frustration because the language they employ in the classroom is not their or their students’ mother tongue (Resnik and Dewaele, 2020). To counteract the negativity and create an instructional and productive learning condition, EFL teachers need to strategically navigate these emotions so as to guarantee the teaching outcomes for students as well as for their career success. The existing studies in the realm of teacher emotion regulation have demonstrated the significant contributions of teaches emotion regulation to successful and effective teaching. Pan and Zeng (2022) investigated the emotion regulation effects on high school teachers and regarded it as a continuous process. The finding suggested teachers employing contextual emotion regulation strategies had increased levels of confidence and more controlling power over stress. In another research by Chang and Taxer (2021), the connection between teacher emotion regulation strategies and classroom misbehavior was investigated. In their study, the regulatory strategies were classified as two streams namely reappraisal and suppression, with the former being more effective when confronting negative emotions in cases of irritative occurrences. Besides the consequences, the possible associates and contributing constructs to emotion regulation have been researched. Jin and Li (2023) analyzed the challenge of emotional management for primary school teachers in China under the “double reduction” policy, which requires to reduce assignments as well as burden for students. The study provided improvement strategies like mobilizing teachers’ emotional management initiative and constructing external support systems. In like manner, Deng et al. (2022) probed in the casual relationship among teacher emotion regulation, self-efficacy beliefs, engagement, and anger. The research was administered to 581 EFL teachers in Iran and the results indicated that emotion regulation could positively and significantly predict teachers’ self-efficacy as well as work engagement. According to a meta-analysis conducted by Wang et al. (2023), teachers’ emotion regulation is influenced by various factors such as environmental, personal, instructional, and well-being factors. The study found that antecedent-focused strategies demonstrated more adaptive associations with the related factors than response-focused strategies. Teachers who receive school support, have engaged and disciplined students, and possess favorable personal characteristics (e.g., conscientiousness) tend to adopt antecedent-focused emotion regulation; these teachers also have greater well-being. In contrast, teachers who work at unsupportive schools or who have relatively unfavorable personal characteristics (e.g., neuroticism) tend to use response-focused strategies; these teachers also have poor teaching effectiveness and well-being.

Research in the domain of teacher emotion regulation still calls for more attention, especially in the quantitative facets. The popular teacher emotion measurement instruments include the “Emotion Regulation Questionnaire” (Gross and John, 2003) and the “Teacher Emotion Regulation Scale” (Buri’c et al., 2017). The former targeted at the general public instead of within teaching context and only focused on two broad perspectives: cognitive reappraisal and expressive suppression, whereas the latter one is more detailed and accurately devoted to teaching environment. It consists of five dimensions: avoiding the situation, active modification strategy, reappraisal, suppression and tension reduction. Developed from the two measurements, Heydarnejad et al. (2021) devised a more psychometrically sound instrument called Language Teacher Emotion Regulation Inventory (LTERI), which added in the dimension of seeking social support into the scale.

## 3 Methodology

This study aimed to examine the correlation and causality among teaching self-efficacy, emotion regulation, and work engagement to gain perspectives of Chinese EFL college teachers’ work engagement for the benefit of in-service teachers as well as for teacher educators. Bear this in mind, a quantitative methods design was adopted to provide a systematic and structured approach to data collection and analysis, allowing researchers to quantify variables and establish measurable relationships between them. By employing statistical tools, quantitative research can handle large datasets, test hypotheses with a high degree of certainty, and offer replicable results that contribute to the cumulative knowledge in a field.

### 3.1 Participants

To maximize the reliability of quantitative data, the author expanded the quantitative data collection period to 4 weeks and adopted the non-probability sampling procedure due to the consideration of feasibility in regard with the restriction of time and cost. Snowball sampling procedure was used in the current study with purposeful selection of participants in terms of accessibility and convenience. Participants (*N* = 495) from 5 provinces and regions responded to the survey, among which 53 participants aged below 25, 77 between 25 to 35 ages, 220 between 36 to 45 ages, and 145 participants aged above 45. The majority of participants aged between 36 and 45 (44.44%). Among the participants, 183 were male (36.97%) and 312 were female teachers (63.03%). More detailed demographic information about participants is listed in Table 1.

**Table 1:** Demographic statistics.

Items	Frequency	Percent (%)
Gender		
Male	183	36.97
Female	312	63.03
Age group		
Below 25	53	10.70
Between 25 and 35	77	15.56
Between 36 and 45	220	44.44
Above 45	145	29.30
Education background		
Bachelor of Arts	115	23.23
Master of Arts	302	61.01
PhD	78	15.76
Years of teaching		
Less than 5 years	82	16.57
5 to 10 years	85	17.17
11 to 15 years	115	23.23
16 to 20 years	113	22.83
Over 20 years	100	20.20

### 3.2 Instruments

The study used three established measuring scales to explore the interrelationships between EFL teachers’ emotion regulation, teaching self-efficacy and work engagement, details of which are listed in Table 2.

**Table 2:** Established measuring scales used in the study.

Measuring variables	Measuring scale	Factors	Item number	Source
Emotion regulation	Language Teacher Emotion Regulation Inventory (LTERI)	Situation Selection	5	Heydarnejad et al. (2021)
		Situation Modification	5	
		Attention Deployment	4	
		Reappraisal	5	
		Suppression	4	
		Seeking Social Support	4	
Teaching self-efficacy	Teacher Efficacy Scale	General Teaching self-efficacy	5	Hoy and Woolfolk (1993)
		Personal Teaching self-efficacy	5	
Teachers’ work engagement	The Engaged Teacher Scale (ETS)	Emotion Engagement	4	Klassen et al. (2012)
		Social Engagement: Colleagues	4	
		Cognitive Engagement	4	
		Social Engagement: Students	4	

Designed on the basis of the process model of emotion regulation (Gross, 1998), Heydarnejad et al. (2021) devised a more psychometrically sound instrument called Language Teacher Emotion Regulation Inventory (LTERI), which took the manner of teachers’ socialization into consideration and added in the dimension of seeking social support into the scale. LTERI scale has been well-tested in many relevant research under different culture context and has been considered a valid and widely-used self-report instrument for the measurement of language teachers’ emotion regulation (Ma, 2023; Namaziandost et al., 2023), thus this measurement scale was adopted for this study to evaluation the construct of teachers’ emotion regulation. It includes 27 items on a 5-point Likert scale anchored by 1 (‘never’) to 5 (‘always’), targeting at six factors, namely situation selection, situation modification, attention deployment, reappraisal, suppression, and seeking social support. The internal consistency and reliability of the scale were assessed through Cronbach’s alpha coefficient and the result was acceptable (ranging from 0.786 to 0.866).

Developed from the Teacher Efficacy Scale (Gibson and Dembo, 1984), the short form of the Teacher Efficacy Scale (Hoy and Woolfolk, 1993) probed teachers’ teaching self-efficacy from two dimensions (i.e., general teaching self-efficacy and personal teaching self-efficacy) with 10 items in total. Example GTE items is ‘If students aren’t disciplined at home, they aren’t likely to accept any discipline.’ and example PTE items is ‘If a student did not remember information I gave in a previous lesson, I would know how to increase his/her retention in the next lesson.’ Response to each item is along a 5-point Likert scale from ‘strongly disagree’ to ‘strongly agree’. The items in this scale were reported had high factor loadings and its reliability was justified by Cronbach’s alpha ranging from 0.881 to 0.906.

Teachers’ work engagement was assessed by the Engaged Teacher Scale (ETS) (Klassen et al., 2012). This instrument included 16 items and it was adapted to be on a 5-point Likert scale (1 representing ‘strongly disagree’ and 5 representing ‘strongly agree’) in this research to match the other two scales, with four factors consisting of emotional engagement, college social engagement, cognitive engagement, and student social engagement. The reliability of the scale was reported acceptable for both sub-components with Cronbach’s alpha of 0.780 and 0.818.

Although all respondents were EFL teaches in college and they enjoyed advanced English proficiency levels, the above-mentioned measuring scales were translated into Chinese (participants’ native language) by three experienced Chinese EFL teachers (with at least 15 years of teaching) to make participants feel more comfortable and agreeable in the survey. The translated version was then subjected to a preliminary pilot test involved a sample of 20 EFL teachers, and the results confirmed the scale’s internal consistency (Cronbach’s alpha > 0.85) and factor structure. To prevent customary response from participants and achieve more reliable results, the items of three different scales were integrated then interspersed randomly thus formed an all-inclusive questionnaire containing 53 items. In addition, the demographic information of participants was collected at the beginning of questionnaire, including their gender, age, academic degree, and years of teaching. The finalized version with all 57 items was uploaded onto the online platform Survey Star for the distribution of questionnaire.

### 3.3 Procedure

This research project adopted the non-probability sampling procedure due to the consideration of feasibility in regard with the restriction of time and cost. Snowball sampling was also used in this study with purposeful selection of participants in the overall population in terms of investigation characteristics, accessibility, and convenience (Cohen and Arieli, 2011). Once the survey was finalised and uploaded on the Survey Star (wjx.cn), a correspondent QR code generated to be passed on to the potential participants. The survey was first passed onto the researcher’s colleagues and then the colleagues were invited to pass the survey onto other college EFL teachers via various social networking applications such WeChat. It was announced explicitly that the survey was completely voluntary and anonymous, with all participant’ information under confidentiality and used solely for research purpose. The responses with certain features were screened and excluded, including short completion time (less than 100 s) and patterned responses for all items. On account of the design of the electronic survey, all responses had complete demographic variables and no data were missed. The total number of questionnaire responses was 513, among which 18 responses were deemed invalid after filtering, leaving 495 valid responses with an effective response rate of 96.5%.

### 3.4 Data processing

The harvested quantitative data were then submitted to SPSS 26.0 and AMOS 21.0 to analyze the survey reliability, the correlation between the three factors and Structural Equation Model was utilized to explore the predictability power between the constructs.

## 4 Results

The collected data was fed to SPSS software for the evaluation of reliability required for running pertinent statistical techniques. With the preliminary demands met, descriptive statistics and correlation of each construct were demonstrated, answering the first research question and revealing the general picture of the data. Aiming at answering the second research question, regression analysis and SEM were employed.

The sample consisted of teachers with varying ages, teaching experience, and educational backgrounds. Most of the participants were between 36 and 45 years old (44.4%) and had more than 20 years of teaching experience (20.2%). The gender distribution was predominantly female (63.0%), with a significant portion holding a graduate degree (61.0%).

The study employed Cronbach’s alpha to measure the internal consistency and assess the reliability of the three scales with values above 0.70 considered acceptable (Cohen, 1988). All scales demonstrated acceptable reliability, with Cronbach’s alpha values of 0.875 for LTERI, 0.933 for ET, and 0.826 for TSE scale. The detailed Cronbach’s alpha values for each scale and included items were listed in Table 3.

**Table 3:** Reliability analysis.

Scale (Cronbach’s alpha)	Dimension (Cronbach’s alpha)
LTERI (Emotion Regulation) (0.875)	Situation Selection (0.860)
	Situation Modification (0.860)
	Attention Deployment (0.863)
	Reappraisal (0.866)
	Suppression (0.786)
	Seeking Social Supporting (0.815)
ETS (Work Engagement) (0.933)	Emotional Engagement (0.902)
	Social Engagement: Colleagues (0.881)
	Cognitive Engagement (0.891)
	Social Engagement: Students (0.906)
TS (Teaching self-efficacy) (0.826)	General teaching self-efficacy (0.780)
	Personal teaching self-efficacy (0.818)

The descriptive statistics presented in Table 4 provide a comprehensive overview of the central tendencies, dispersion, and shape of the distribution for three key variables in the study, that is emotion regulation (ER), work engagement (WE), and teaching self-efficacy (TSE). The mean (M) values for ER, WE, and TSE are 3.37, 3.58, and 3.49 respectively, indicating that the sample’s central tendency for these variables is slightly above the midpoint of the scales used, which could imply a moderate level of emotion regulation, teacher engagement, and teaching self-efficacy within the sample of teachers. The standard deviation (SD) values, being 0.55 for ER, 0.83 for WE, and 0.74 for TSE, reflecting the degree of variability within the scores, with WE showing the highest variability and TSE the least.

**Table 4:** Descriptive analysis (*N* = 495).

Inventory	Min.	Max.	*M*	*SD*	Skewness (SE)	Kurtosis (SE)
ER	1.00	5.00	3.37	0.55	0.16 (0.11)	−0.46 (0.22)
WE	1.00	5.00	3.58	0.83	0.06 (0.11)	−1.18 (0.22)
TSE	1.00	5.00	3.49	0.74	0.22 (0.11)	−0.30 (0.22)

In assessing the normality of the data, it fell within the accepted benchmarks of −0.5 to 0.5 for skewness and −2 to 2 for kurtosis, indicating an approximately normal distribution (Cohen, 1988). As for skewness values, ER and TSE showed a slight positive skew (0.16 and 0.22, respectively), and WE a slight negative skew (0.06). This suggested that the data for these variables were symmetrical, although there might be a small tendency for extreme values below the mean for ER and TSE, and above the mean for WE. The kurtosis values for ER (−0.46) and TSE (−0.30) indicate a distribution that is less peaked than the normal distribution, suggesting that the data are more spread out along the scale. Conversely, the kurtosis for WE (−1.18) suggest a more pronounced peak and thinner tails, indicating that the data are more concentrated around the mean.

To answer the first research question, Pearson product–moment correlations was run to determine the correlation between constructs and the results were shown in Table 5, indicating a strong positive correlation between emotion regulation (ER) and work engagement (WE) (*r* = 0.698, *p* < 0.01), suggesting that better emotion regulation is associated with higher teacher engagement. Besides, a moderate positive correlation was observed between teaching self-efficacy (TSE) and both ER (*r* = 0.629, *p* < 0.01) and WE (*r* = 0.649, *p* < 0.01), indicating that higher teaching self-efficacy is linked to better emotion regulation and greater teacher engagement.

**Table 5:** Correlations between constructs (*N* = 495).

Inventory	LTERI	ETS	TS
ER	1		
WE	0.698**	1	
TSE	0.629**	0.649**	1

**p< 0.01.

In order to establish structural equation model, the following fit indices were employed to check the model fit. As Jöreskog (1990) explained, “the chi-square should be non-significant, the chi-square/df ratio should be lower than 3, and the root mean square error of approximation (RMSEA) should be lower than 0.1” (p. 12). Based on this standard, the model demonstrated figures with CMIN/DF (Chi-square to degrees of freedom ratio) being 1.262, and the RMSEA was 0.023, both well below the acceptable threshold, indicating an excellent model fit. The SEM analysis was then conducted after ensuring the data’s reliability as well as suitability for such a model.

The second research question was then being answered by examining the strengths of causality. As demonstrated in Table 6, the path from TSE to ER was significant (*p* < 0.05) with a path coefficient of 0.905, thus confirming the hypothesis that teaching self-efficacy positively influences emotion regulation. The correlations between ER and WE were not significant (*p* = 0.471 > 0.05), leading to the failure to confirm the hypothesis that emotion regulation positively affects work engagement. The correlations between TSE and WE were significant (*p* < 0.05) with a path coefficient of 0.745, supporting the hypothesis that teaching self-efficacy positively impacts work engagement.

**Table 6:** Path coefficients, *p*-values, and hypothesis testing results.

Affecting path	Path coefficient value	*p*-value	Hypothesis testing results
TSE → ER	0.905	***	Hypothesis Verified
ER → WE	0.195	0.471	Hypothesis Not Verified
TSE → WE	0.745	0.010	Hypothesis Verified

***p< 0.001.

**Figure 2:**
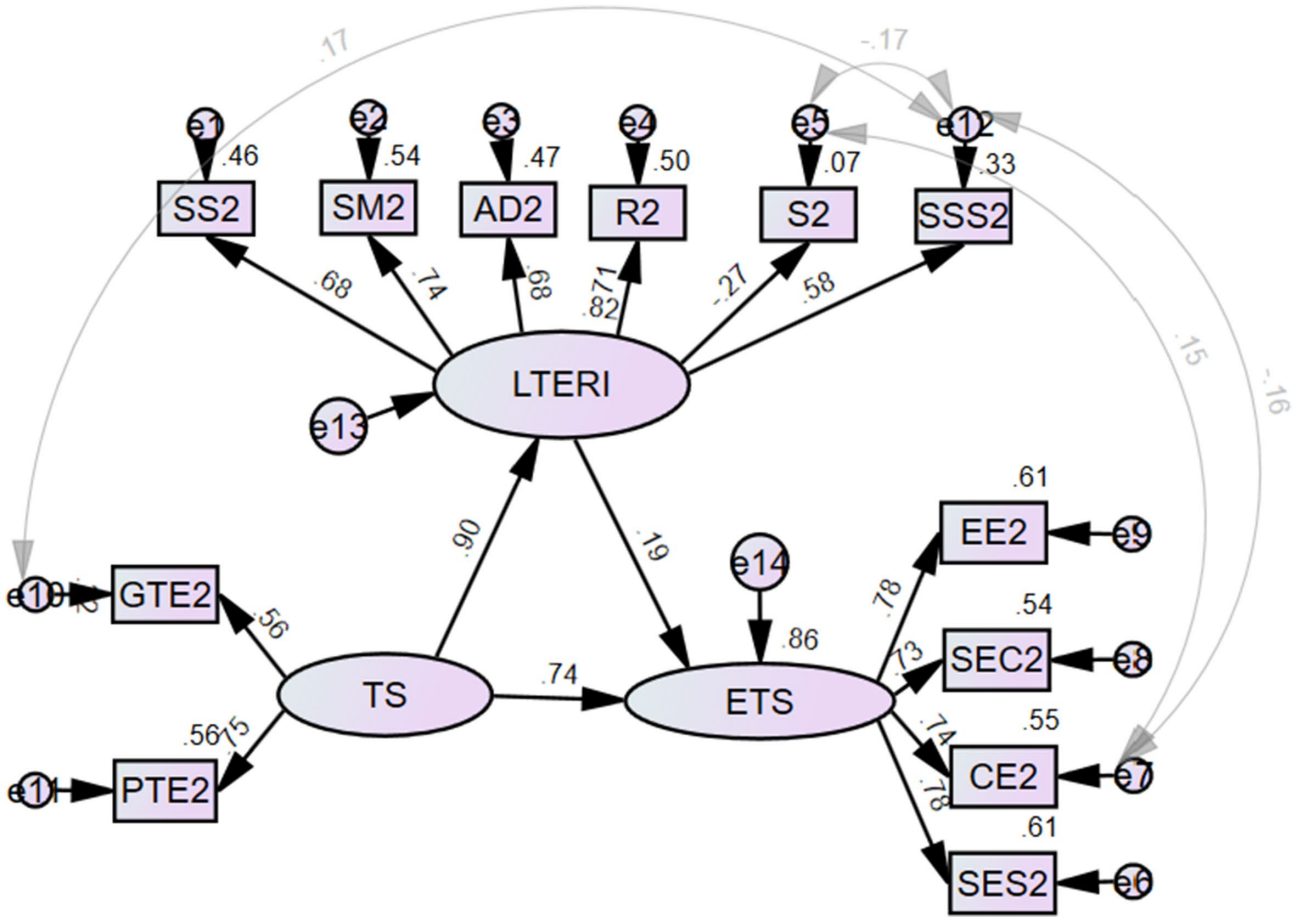
Structural equation model of three constructs. LTERI scale evaluates emotion regulation; TS scale evaluates teaching self-efficacy; ETS scale evaluates work engagement.

As clearly depicted in Figure 2, for emotion regulation scale (LTERI), the most significant positive causality relation was demonstrated by situation modification (SM) with the *β* value of 0.74 while the item of suppression (S) showed a negative relation with the β value of −0.27. The sub-components of teaching self-efficacy scale (TS) and work engagement scale (ETS) all demonstrated positive causality relations with β value ranging from 0.56 to 0.78. Most importantly, according to the SEM, Chinese college EFL teachers’ teaching self-efficacy significantly contributed to their work engagement (*β* = 0.74) as well as emotion regulation (*β* = 0.90), however, emotion regulation failed to predict the work engagement based on the current research data with the β value being 0.19.

## 5 Discussion

The current study aimed to probe into the correlation and causality among teacher emotion regulation, teaching self-efficacy and work engagement to suggest effective strategies to enhance EFL teachers’ work engagement for the benefit of in-service teachers as well as for teacher educators. The gained results from Pearson’s correlation analysis clearly answered the first research question and revealed strong positive relationship between the participants’ emotion regulation and work engagement and moderate positive relationship between teaching self-efficacy and work engagement. The findings supported the previous studies disclosing that there was intertwined correlation between emotion regulation, teaching self-efficacy and teachers’ work engagement (Deng et al., 2022; Han and Wang, 2021), indicating that teachers’ ability to adjust their emotion and their self-recognition competence were closely associated with their dedication and commitment in the educational context.

Additionally, the results also suggested a significant positive relationship between teaching self-efficacy and emotion regulation, indicating that teachers who felt more efficacious in their teaching abilities also exhibited better emotion regulation skills. This finding was in line with that of Deng et al. (2022) who identified positive correlation between emotion regulation and self-efficacy. This could be explained by the fact that teaches with higher self-efficacy would deem themselves as competent in the teaching context, having acquired sufficient knowledge and methodologies so as to prevail over changes and challenges, therefore, they tend to maintain a positive mentality and higher level of grittiness under the situation of adversity. On the other hand, teaching self-efficacy was also found to have a direct positive impact on work engagement, which aligned with the literature that suggested confident teachers are more likely to be engaged in their work, demonstrating a sense of commitment and vigor in their teaching practices (Wang and Pan, 2023).

In order to answer the second research question regarding the predicting power of emotion regulation and teaching self-efficacy on work engagement, SEM was established and the result in the current study demonstrated that teaching self-efficacy had strong positive predictability on work engagement as well as emotion regulation, claiming that teachers with high level of self-confidence and self-acceptance would show more capability in managing their emotional ups and downs, thus in turn being more committed to their teaching practice and positive outcomes were more readily obtained. The results were consistent with the findings of Fathi et al. (2020), who claimed that teaching self-efficacy was a pronounced predictor among positive psychological factors on emotion regulation as well as on work engagement, and they argued that teachers with higher level of self-efficacy in their teaching practice would demonstrate more endeavor to overcome challenges or exhibit more resilience when encountering emotional frustrations and turbulences.

However, as a surprising finding, the result of current study did not find support for the hypothesis that emotion regulation would constructively enhance work engagement. This particular finding contradicted with many previously published research results done in educational context, which claiming emotion regulation could positively influence and enhance work engagement for EFL teachers (Chen and Tang, 2024; Greenier et al., 2021). However, the study done by Xie (2021) exhibited a similar conclusion, which also discovered that as two major components of emotional regulation, cognitive reappraisal and expressive suppression failed to predict teachers’ work engagement. A logic behind this discrepancy could be due to various factors not accounted for in the model that might influence work engagement, such as unique Chinese culture, professional expertise or autonomy, personal values and motivation.

The findings of this study indicates that educators with a heightened sense of self-efficacy are likely to experience an enhanced confidence or an increased sense of self-assurance in their pedagogical abilities. This emotional self-efficacy can lead to a more assertive and proactive approach to classroom management, as teachers who feel they are in command of their emotional ups and downs are better equipped to handle the diverse emotional landscapes that emerge in educational settings. By navigating the complexities of the classroom with greater confidence and competence, teachers can create positive emotional bonding between students and themselves, in turn student engagement can be better fostered thus enhancing their learning outcomes. What is more, in some challenging teaching contexts such as low interaction rate or diverse learning needs, educators with stable emotion states and intricate emotional management strategies can be better positioned to address student behavioral issues calmly and reasonably and to provide constructive feedback.

## 6 Conclusion

The present study provided empirical evidence that teaching self-efficacy is a key predictor of both emotion regulation and work engagement among Chinese EFL teachers. The results highlighted the need for educational policies and professional development programs that supported teachers in developing their teaching self-efficacy skills. By putting emphasis on enhancing teaching self-efficacy, college can foster a more engaged and effective teaching workforce, ultimately contributing to improved educational experiences and outcomes for students.

On the other hand, while emotion regulation did not directly affect work engagement in this model, it remained an in influential area for future research, especially considering the complex emotional landscape of teaching and unique Chinese culture. The findings of current study underscored the essential driving power of situation modification and reappraisal as two major contributing items to teachers’ emotion regulation, as well as the negative predictive force of suppression.

This study provides some pedagogical implications for in-service educators and administrators in regard with the strategies to enhance work engagement. Firstly, educators in university can benefit from the findings by becoming aware of and putting emphasis on their psychological constructs such as self-efficacy to achieve better performance in their career development. EFL teachers are expected to raise their awareness of the value of emotion regulation strategies in the process of their classroom instructions and need to be more efficient in handling their emotional experiences. As an EFL instructor, one needs to regard the behavior of emotion regulation as an art, with the proper use of antecedent-focused strategies to take actions before an emotional response has fully developed, and response-focused strategies, which are employed after an emotional response has occurred. For example, avoiding those situations which are likely to provoke negative emotions is regarded as an antecedent-focused strategy while consciously hiding one’s facial expressions or body language is considered as a response-focused strategy.

On the other hand, university administrators, with their crucial role in fostering a dynamic and motivated faculty, may gain some perspectives from the study results as to how to promote university teachers’ enthusiasm in work. By examining the study results that highlight the importance of teaching self-efficacy and emotion regulation, they can develop targeted initiatives to boost teacher enthusiasm and work engagement. For example, seeing the essential role of teaching self-efficacy in enhancing work engagement, university authority can organize psychological workshops or provide profession guidance for in-service teachers regularly to facilitate them with their emotional or psychological nuisances.

## 7 Limitations

The current study had some limitations which can be the target of improvement for future researchers. In the study, cross-sectional design was adopted, limiting the ability to draw conclusions about causality in the long-run, therefore, future research could employ longitudinal designs to better understand the directionality of the relationships between teaching self-efficacy, emotion regulation, and work engagement. Additionally, the generalizability of the findings may be limited by the specific context of Chinese EFL education, thus cross-cultural studies could provide further insight into the universality of a wider range. What’s more, future studies could explore other potential predictors of work engagement, such as school leadership, peer support, and professional development opportunities. Lastly, as quantitative research design was adopted in this study, qualitative research method could be implemented to unveil a deeper understanding of the experiences and challenges encountered by EFL teachers in Chinese higher education context.

## Data Availability

The raw data supporting the conclusions of this article will be made available by the authors, without undue reservation.
